# Transcriptome-wide N6-methyladenine methylation in granulosa cells of women with decreased ovarian reserve

**DOI:** 10.1186/s12864-022-08462-3

**Published:** 2022-03-28

**Authors:** Chang Liu, Linshuang Li, Bo Yang, Yiqing Zhao, Xiyuan Dong, Lixia Zhu, Xinling Ren, Bo Huang, Jing Yue, Lei Jin, Hanwang Zhang, Lan Wang

**Affiliations:** 1 Reproductive Medicine Center, The Affiliated Drum Tower Hospital of Nanjing University Medical School, Nanjing, People’s Republic of China; 2 Reproductive Medicine Center, Tongji Hospital, Tongji Medical College, Huazhong University of Science and Technology, Wuhan, People’s Republic of China; 3 Institute of Organ Transplantation, Tongji Hospital, Tongji Medical College, Huazhong University of Science and Technology, Wuhan, People’s Republic of China

**Keywords:** Granulosa cell, N6-methyladenosine, RNA modification, Aging, Ovary

## Abstract

**Background:**

The emerging epitranscriptome plays an essential role in female fertility. As the most prevalent internal mRNA modification, N6-methyladenine (m^6^A) methylation regulate mRNA fate and translational efficiency. However, whether m^6^A methylation was involved in the aging-related ovarian reserve decline has not been investigated. Herein, we performed m^6^A transcriptome-wide profiling in the ovarian granulosa cells of younger women (younger group) and older women (older group).

**Results:**

m^6^A methylation distribution was highly conserved and enriched in the CDS and 3’UTR region. Besides, an increased number of m^6^A methylated genes were identified in the older group. Bioinformatics analysis indicated that m^6^A methylated genes were enriched in the FoxO signaling pathway, adherens junction, and regulation of actin cytoskeleton. A total of 435 genes were differently expressed in the older group, moreover, 58 of them were modified by m^6^A. Several specific genes, including BUB1B, PHC2, TOP2A, DDR2, KLF13, and RYR2 which were differently expressed and modified by m^6^A, were validated using qRT-PCR and might be involved in the decreased ovarian functions in the aging ovary.

**Conclusions:**

Hence, our finding revealed the transcriptional significance of m^6^A modifications and provide potential therapeutic targets to promote fertility reservation for aging women.

**Supplementary Information:**

The online version contains supplementary material available at 10.1186/s12864-022-08462-3.

## Introduction

Ovary is a critical regulator of female fertility, serving as the source of oocytes and a major supplier of steroid sex hormones [1]. As a reproductive organ, ovary exhibits a rate of aging that is much faster than other somatic organs [2]. According to the human biologic clock, female fertility starts at puberty and decreases after the age of 30, with a steep decrease after 35, culminating in the menopause at 50 years of age [3, 4]. With the modern tendency of delaying childbearing, ovarian aging has become an age-related disease that entails careful considerations for reproductive care systems [5, 6].

Ovarian functional decline with aging is generally characterized by the gradual decrease in both the quantity and quality of ovarian follicles [2, 7, 8]. Multiple factors influence oocyte quality during aging [9]. Granulosa cells, which form multiple layers surrounding the oocyte and communicate with oocytes via direct contact or paracrine pathways, play critical roles in regulating oocyte competence [10]. Besides, granulosa cells also provide oocytes with essential nutrients, maintain oocyte arrest, and induce meiosis [11, 12]. Accumulating evidence has confirmed that aging increases oxidatively damaged lipids, proteins, and DNAs in the granulosa cells [5, 13]. Moreover, aged granulosa cells may inhibit oocyte potentials, since removed from the follicular microenvironment and matured *in vitro*, oocytes could be partially protected from age-related defects [14]. Therefore, characterizing the changes in the aged granulosa cells might enable the mechanisms of diminished ovarian functions of aging to be elucidated.

As tissues or cells get old, the proteins they produce elicit a senescence-associated phenotype [15]. These changes in gene expression phenotypes are driven by epigenetic modifications. Studies have shown that oocytes of donors with increased maternal age exhibited aberrant global DNA methylation levels [16]. Moreover, the abundance of histone H4K12 acetylation was correlated with increased misalignment of chromosomes in the oocytes of older women [17]. Other than DNA and histone modification, emerging evidence suggests that RNA modification, especially N6-methyladenine (m^6^A), is one of the most prevalent epigenetic modifications affecting the regulation of gene expression [18]. It has been well established that m^6^A modifications are essential for folliculogenesis, oocyte development, and maturation [19–21]. Besides, recent evidence indicated that m^6^A demethylase was decreased in the aged ovaries, suggesting that m^6^A methylation might participate in the process of ovarian aging [22]. However, whether m^6^A methylation is associated with the diminished ovarian reserve in the aged ovaries, has not been clarified.

Therefore, in the present study, we aim to characterize the m^6^A epigenetic profiles and their functions in the granulosa cells of naturally aging women. Granulosa cells collected from older women and younger women were used for m^6^A-targeted antibody coupled with high-throughput sequencing (MeRIP-Seq) and RNA sequencing (RNA-Seq). The potential functions of the modified genes were analyzed and validated using quantitative real-time polymerase chain reaction (qRT-PCR).

## Material and methods

### Study population

A total of twelve women were recruited in the study. Since ovarian function was gradually decreased from the mid to late 30 s [23], women aged 25-30 years and presented with a normal ovarian reserve (antral follicular count (AFC)>=5; anti-müllarian hormone (AMH)>=1.2 ng/mL) and normal ovarian response (number of retrieved oocytes>=9), were recruited in the younger group. Besides, these women appeal to *in vitro* fertilization (IVF) treatments because of tubal or male factors. Meanwhile, women aged between 40 years and 50 years, presented with a diminished ovarian reserve (AFC<5; AMH<1.2 ng/mL) or poor ovarian response (number of retrieved oocytes<9) [24, 25] were recruited in the older group. The exclusive criteria were uterine abnormalities, endometriosis, polycystic ovarian syndrome, diabetes, thyroid diseases, hyperprolactinemia, karyotype anomalies, repeated spontaneous abortion, primary infertility over 5 years, and unexplained infertility. All participants had normal body mass index (BMI, ranged 18.5-24) and received IVF treatment with GnRH antagonist protocols. Granulosa cells were collected at the time of oocyte retrieval. Women were totally informed of the procedures, and signed informed consent was obtained from all participants. This study was approved by the Institutional Review Board of Tongji Hospital (TJ-IRB20210213).

### Granulosa cell collection

Follicular fluid aspirated from follicles of individuals during oocyte retrieval was pooled and considered as independent samples. Granulosa cells were isolated from the follicular fluid as previously described [26]. Briefly, follicular fluid was centrifugated at 1200 rpm for 10 min to precipitate cells. The cell pellets were resuspended in PBS and layered onto a 50% Percoll Reagent (Sigma-Aldrich, MO, USA) and centrifugated at 1200 rpm for 30 min to separate granulosa cells from blood cells. Granulosa cells at the interface were harvested and washed with PBS and red blood cell lysis buffer (Servicebio, Wuhan, China). After centrifugation at 1500 rpm for 15 min, granulosa cells were collected and used for RNA extraction.

### m^6^A-targeted antibody coupled with high-throughput sequencing (MeRIP-Seq) and RNA sequencing (RNA-Seq)

Three pairs of samples from the two groups were used for MeRIP-Seq and RNA-Seq, while other samples were used for validation. The MeRIP-Seq was performed in accordance with the published procedure with slight modifications. Briefly, the total RNA of granulosa cells was extracted using RNA-easy Isolation Reagent (Vazyme, Nanjing, China) following the manufacturer’s instructions. The quality of extracted RNA was determined using Nanodrop spectrophotometer for RNA purity (A260/A280) and standard denaturing agarose gel electrophoresis for RNA integrity. RNA was randomly fragmented to 200 nt and purified with the RNA clean and concentrator kit (Zymo Research, CA, USA). A total of 1 μg fragmented mRNA was used for RNA-Seq, while 500 μg of fragmented RNA was incubated with anti-m^6^A polyclonal antibody (ABE572, Sigma-Aldrich, MO, USA), followed by immunoprecipitation with protein-A beads (10002D, Invitrogen, CA, USA) and protein-G beads (10004D, Invitrogen, CA, USA). Bound RNA was extracted with TRIzol reagent (15596018, Invitrogen, CA, USA). Both the m^6^A immunoprecipitated samples and the input samples were used for library construction with Illumina NovaSeq 6000 sequencer (Illumina, CA, USA).

### Data analysis

The quality of the raw reads was determined by FastQC. Trimming was performed for known Illumina TruSeq adapter sequences, poor reads, and ribosomal RNA reads. Then, clean reads were aligned to the human reference genome with the use of Hisat2 software (v2.0.4). Methylated sites on RNAs were identified by MACS software. Cutadapt (v2.5 ) was used to trim adapters and filter for sequences, remaining reads were then aligned to the human Ensemble genome GRCh38 (mouse Ensemble genome GRCm38) using Hisat2 aligner (v2.1.0) under parameters: “-rna-strandness RF”. M^6^A peaks were identified using exomePeak R package (v2.13.2) under parameters: “PEAK_CUTOFF_PVALUE = 0.05, PEAK_CUTOFF_FDR = NA, FRAGMENT_LENGTH = 200”. Differential m^6^A peak were identified using exomePeak R package under parameters: “PEAK_CUTOFF_PVALUE = 0.05, PEAK_CUTOFF_FDR = NA, FRAGMENT_LENGTH = 200”. Gene ontology (GO) and Kyoto Encyclopedia of Genes and Genomes (KEGG) pathway enrichment analysis were performed using clusterprofile R package (v3.6.0) [27–29]. M^6^A-RNA-related genomic features were visualized using Guitar R package (v1.16.0). Identified m^6^A peaks which adjusted *p* value <0.05 were chosen for the de novo motif analysis using homer (v4.10.4) under parameters: “-len 6 -rna”. The reads mapped the genome were calculated using featureCounts (v1.6.3). Differential gene expression analysis was performed using the DESeq2 R-package. Differentially methylated sites with a |fold change| cutoff of >2 and an adjusted *p* value of <0.05 were identified by diffReps. Gene set enrichment analysis (GSEA) [30] was performed to further explore signaling pathways and cellular functions in the process of female reproductive aging.

### RNA extraction and qRT-PCR

RNA extraction and qRT-PCR were performed following the published procedures with minor modifications [31, 32]. Briefly, RNA of granulosa cells was extracted using RNA-easy Isolation Reagent (Vazyme, Nanjing, China) following the manufacturer’s instructions. Then, 500 ng RNA was used for cDNA synthesis with the use of PrimeScript™ RT Master Mix (RR036A, Takara, Japan). qRT-PCR was performed with SYBR® Premix Ex Taq™ II (RR420A, Takara, Japan) and Light Cycler 96 instrument (Roche, Mannheim, Germany). Each sample was detected in triplicate and the relative gene expression was normalized to GAPDH. The oligonucleotide sequences of primers used in the experiments were listed in Table 5.

### Statistical analysis

Quantitative variables were expressed as mean ± standard deviation (SD), and Student’s *t*-test was performed to evaluate statistical significance. Statistical significance was set at the adjusted *p*-value < 0.05.

## Results

### Clinical characteristics of participants

The clinical characteristics of samples that used for MeRIP-Seq was listed in Table 1. Besides, the detailed controlled ovarian stimulation protocols for individuals were displayed in the Supplemental Table 1. As data indicated, the age of the younger group was significantly different from that in the older group (*p*<0.001). Besides, AFC was significantly decreased in the older group compared to the younger group (*p *= 0.001). No statistical significance was found in the menstrual cycle and basal hormonal levels between the two groups.

**Table 1:** Clinical characteristics of participants.

Variables	younger (*n* = 3)	older (*n* = 3)	*p*-value
Age (years)	26.33 ± 1.15	43.67 ± 1.15	<0.001
Menstrual cycle (days)	30.33 ± 2.52	22.61 ± 1.51	0.10
FSH (mIU/mL)	9.04 ± 3.11	7.26 ± 4.04	0.58
LH (mIU/mL)	6.13 ± 3.01	3.75 ± 2.30	0.34
AMH (ng/mL)	2.85 ± 1.66	0.72 ± 0.57	0.10
AFC	13.33 ± 2.08	3.00 ± 1.00	0.001

Data were presented as mean ± standard deviation (SD)

*AFC *antral follicle count

### Overview of m^6^A methylation in the granulosa cell mRNA

**Fig. 1:**
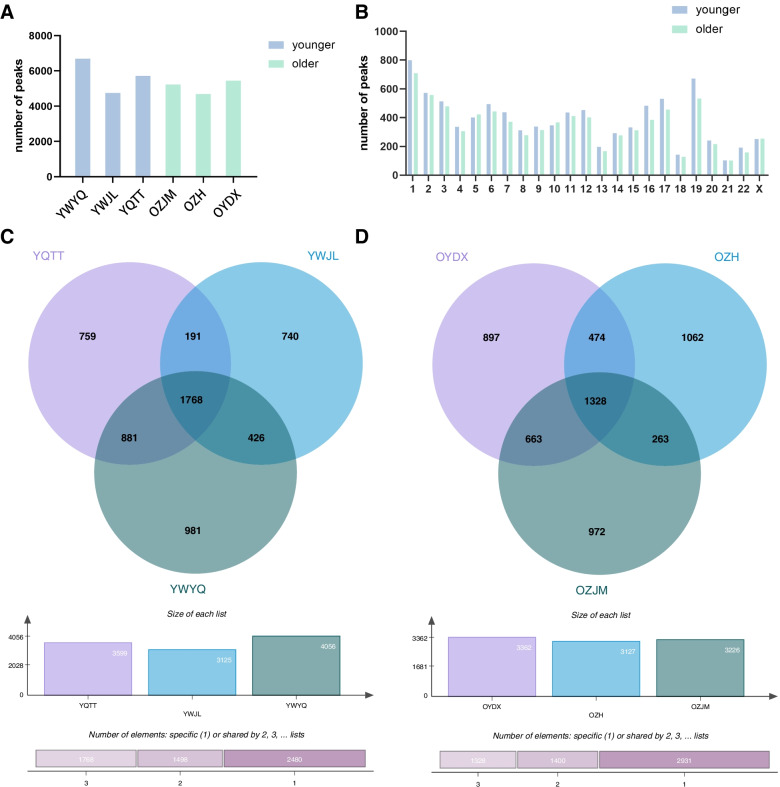
Overview of m^6^A methylation in the granulosa cell mRNA. **A** The number of m^6^A peaks in each sample. **B** The number of m^6^A peaks in each chromosome. **C** Venn diagram of m^6^A peaks in younger groups. **D** Venn diagram of m^6^A peaks in older group. YWJL, YWYQ, YQTT, OZH, OZJM, OYDX are labels of the six participants in the both groups. Y stands for the younger group and O stands for the older group. The rest letters are initials of each participant

Granulosa cells collected from older and younger women were used for MeRIP-Seq to obtain the transcriptome-wide m^6^A map. In the younger group, 6689, 4765, 5719 m^6^A peaks were identified in each sample, respectively. Meanwhile, there were 5243, 4703, and 5449 detected m^6^A peaks in the samples of the older group (Fig. 1A). m^6^A methylation distribution at different chromosome loci was shown (Fig. 1B). Data showed that the most m^6^A methylation were chromosome 1 with 799 m^6^A peaks and chromosome 19 with 671 m^6^A peaks. The Venn diagram was provided in Fig. 1C-D. In the Venn diagram, we displayed the number of m^6^A methylated genes of each sample. In the older group, there were 5147 peaks with read counts more than 0 in sample OYDX and OZH, 4902 peaks with read counts more than 0 in sample OZH and OZJM, 6242 peaks with read counts more than 0 in sample OYDX and OZJM, altogether 4829 peaks with read counts more than 0 in the three samples. In the younger group, there were 5562 peaks with read counts more than 0 in sample in sample YQTT and YWJL, 6739 peaks with reads counts more than 0 in sample YQTT and YWYQ, 5878 peaks with reads counts more than 0 in sample YWJL and YWYQ, altogether 5465 peaks with reads counts more than 0 in the three samples. Averagely, there were 1846 reads in each peak for the older group and 2149 reads in each peak for the younger group, indicating that the number of m^6^A methylation sites was higher in the younger group. Whereas the number of m^6^A methylated genes was higher in the older group (5147 genes) compared to the younger group (3248 genes; See Dataset 1 in the related files)

### Different m^6^A methylation of genes in the granulosa cells of older women

**Fig. 2:**
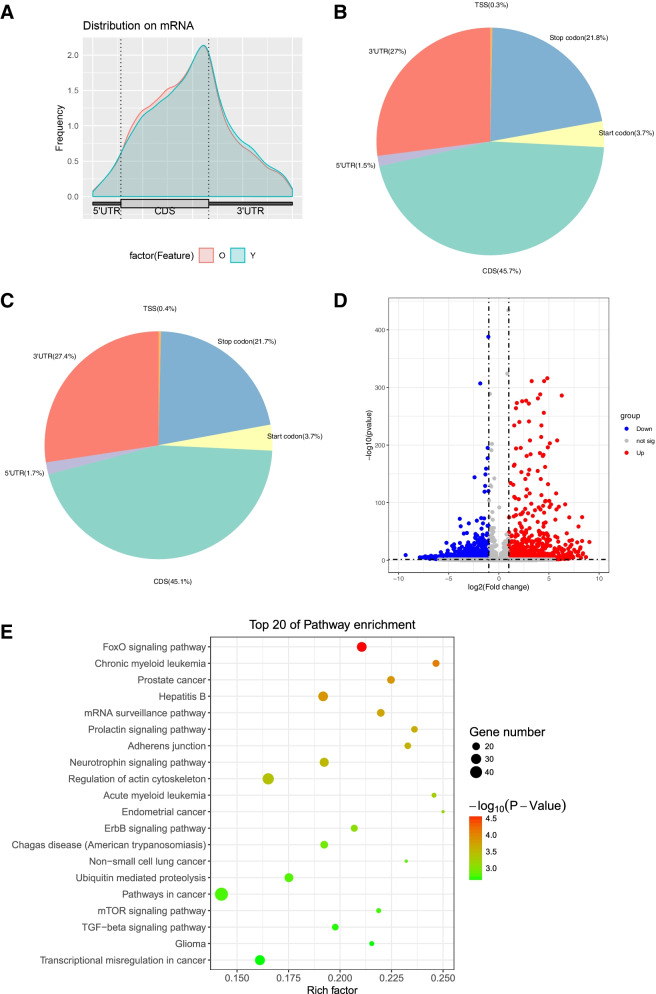
Different m^6^A methylation of genes in the granulosa cells of older women. **A** The distribution of m^6^A peaks across the length of mRNAs were compared in the two groups. **B** The distribution of m6A peaks in the older group. **C** The distribution of m^6^A peaks in the younger group. **D** Volcano plots display the different m^6^A methylation peaks with statistical significance (|fold change|>2 and adjusted *p *< 0.05). Red dots represent the hypermethylated transcripts and blue dots represent the hypomethylated transcripts. **E** The top 20 significantly enriched pathways for the differently methylated genes in the older group

The metagene profiles of different m^6^A peaks for the whole transcriptome visualized m^6^A methylated regions in the two groups. Interestingly, m^6^A peak distributions were similar between the two groups (Fig. 2A). Further analysis calculated the preferential localization of m^6^A on RNA transcripts by dividing m^6^A peaks into six nonoverlapping genic regions: 5’ untranslated region (UTR), transcription start site (TSS), start codon, coding sequence (CDS), stop codon, and 3’ UTR (Fig. 2B-C). Data showed that the most m^6^A methylated peaks were concentrated in the CDS (45.1%-45.7%) and 3’ UTR (27%-27.4%), followed by stop codon (21.7%-21.8%), start codon (3.7%), 5’UTR (1.5%-1.7%), and TSS (0.3%-0.4%). These results showed the highly consistent topological patterns of m^6^A methylation, indicating the conservation of motif recognition of m^6^A methylation in the granulosa cells. Here the motif refers to “GGAC” (Suppl Fig. 1).

The differently m^6^A methylated peaks in the older group were analyzed with the criteria of |fold change|>2 and adjusted *p*-value <0.05 (Fig. 2D). A total of 3005 genes with different m^6^A methylation in the older group compared to the younger group, with 1297 hypermethylated and 1708 hypomethylated. The top 10 hypermethylated and hypomethylated genes in the older group were listed in Table 2. The function of these differently methylated genes was annotated with GO and KEGG pathway analysis. The top 20 enriched Go terms and top 20 pathway enrichments of the differently m^6^A methylated genes were shown. Results indicated that the differently methylated genes were enriched in the regulation of transcription, cellular response to DNA damage stimulus, and apoptosis (Suppl Fig.2). Moreover, the associated pathway of the differently methylated genes including FoxO signaling pathway, adherens junction, and regulation of actin cytoskeleton (Fig. 2E).

**Table 2:** Top ten hypermethylated and hypomethylated genes in the older group

gene	pattern	chromosome	peak region	peak start	peak end	log2.FC	lg.p
PRPF6	Hyper	20	CDS	63984943	63994934	6.78	-46.7
KPNB1	Hyper	17	3’UTR	47683285	47683436	5.82	-208
QKI	Hyper	6	CDS	163478842	163535115	5.72	-116
LARP7	Hyper	4	CDS	112647817	112650501	5.07	-321
ANP32E	Hyper	1	CDS	150220739	150229200	4.72	-35.8
ARID4B	Hyper	1	CDS	235213858	235219954	4.67	-65.6
ICE1	Hyper	5	CDS	5462879	5463030	4.6	-11.8
SCAPER	Hyper	15	Exon	76705922	76764969	4.49	-256
ABCF1	Hyper	6	CDS	30579931	30583093	4.41	-46.5
YLPM1	Hyper	14	CDS	74797722	74798023	4.37	-20.2
PCNT	Hypo	21	CDS	46357108	46363666	-5.22	-30.2
ALB	Hypo	4	CDS	73413418	73415297	-5.17	-11.8
RAB35	Hypo	12	3’UTR	120097090	120097299	-5.11	-6.55
PLLP	Hypo	16	3’UTR	57252248	57252549	-4.75	-9.84
PEAK1	Hypo	15	CDS	77180157	77180548	-4.63	-7.46
GNPAT	Hypo	1	CDS	231267739	231269870	-4.46	-27.6
CAMSAP1	Hypo	9	CDS	135822676	135823006	-4.1	-5.91
FKBP11	Hypo	12	exon	48923428	48923668	-4.04	-14.3
INS-IGF2	Hypo	11	3’UTR	2132317	2132527	-3.95	-19.9
FKBP11	Hypo	12	exon	48923420	48923660	-3.93	-13.8

### Different mRNA expression profiles in the granulosa cells of older women

**Fig. 3:**
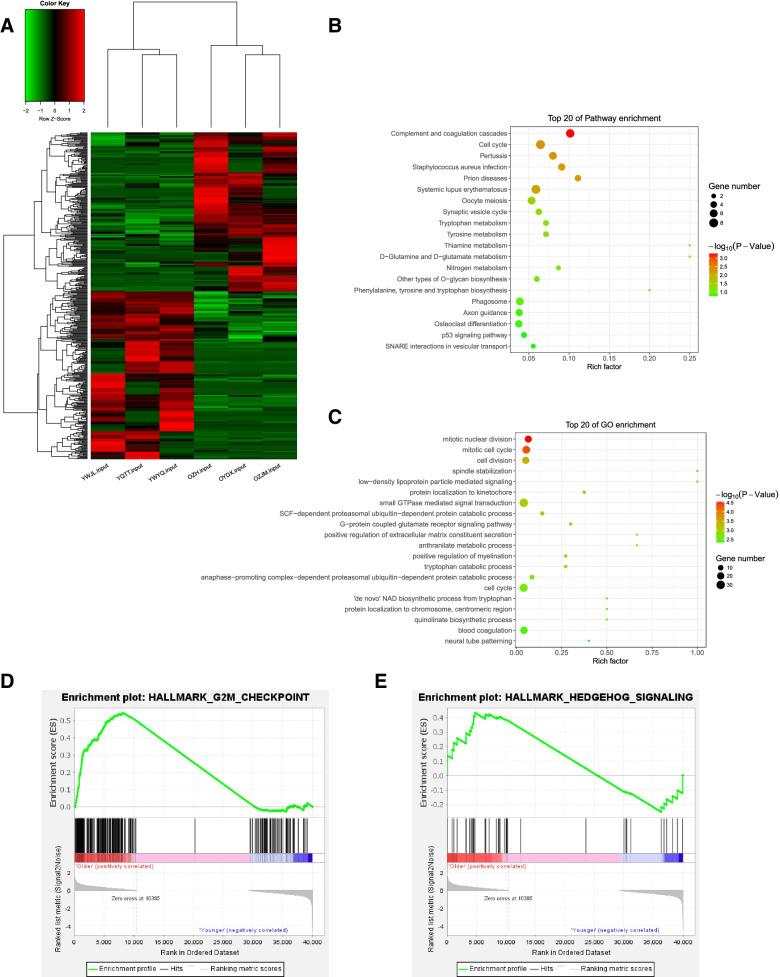
Different mRNA expression profiles in the granulosa cells of older women. **A** Hierarchical cluster analysis of differentially expressed genes between the two groups. **B** The top 20 significantly enriched pathways for the differently expressed genes in the older group. **C** The top 20 enriched GO terms of differentially expressed genes in the older group. GSEA analysis of G2M checkpoint **D** and hedgehog pathways **E**

RNA-Seq revealed a significantly different mRNA expression profile in the granulosa cells of older women compared to younger women. The hierarchical clustering of the mRNA expression was shown (Fig. 3A). A total of 435 differently expressed genes (|fold change|>2 and adjusted *p* < 0.05) were identified, with 212 genes up-regulated and 223 genes down-regulated in the older group. The top 10 up-regulated and down-regulated genes in the older group were listed in Table 3. The functions of the differently expressed genes were analyzed using GO and KEGG pathway analysis. Results showed that the most relevant pathways were complement and coagulation cascades, and cell cycle pathway (Fig. 3B). Moreover, GO enrichment terms indicated the differently expressed genes were enriched in mitotic nuclear division and mitotic cell cycle (Fig. 3C). GSEA analysis of the entire expression profiles indicates various cellular processes and structures affected by female reproductive aging. We found G2M checkpoint pathway (adjusted *p*-value<0.001) and hedgehog signaling pathway (adjusted *p*-value=0.084) were significantly enriched in ovarian granulosa cells of older women (Fig. 3D-E). The detailed information and the collection of genes of core enrichment in each pathway were listed in the supplemental materials (See Dataset 2 in the related files).

**Table 3:** Top ten up-regulated and down-regulated genes in the older group

gene	pattern	log2.FC	lg.p
C2	Up	9.01	-5.72
DNAJC22	Up	8.99	-5.95
GABBR2	Up	8.94	-5.44
HSPB6	Up	8.86	-4.76
NPC1L1	Up	8.80	-5.27
GLP2R	Up	8.78	-3.85
FAM83D	Up	8.42	-3.21
CENPA	Up	8.26	-2.94
GPR21	Up	8.19	-2.80
RASGRF1	Up	8.17	-2.78
CDH13	Down	-8.79	-4.07
NRG1	Down	-8.60	-4.71
SRSF12	Down	-8.35	-3.26
FBXL13	Down	-8.33	-3.86
ADM2	Down	-8.32	-3.22
SMIM10L2B	Down	-8.27	-3.03
SLC16A2	Down	-8.17	-3.96
BPIFB2	Down	-8.02	-2.75
F12	Down	-7.90	-2.55
DPYSL4	Down	-7.90	-2.56

### Relationship between m^6^A methylation and mRNA expression

A conjoint analysis was conducted for m^6^A-seq and RNA-Seq data. We found 58 genes that commonly had different m^6^A methylation levels and differently expressed mRNA levels (Table 4). Among them, 11 genes were up-regulated with hypermethylation, 15 genes were down-regulated with hypermethylation, 21 genes were up-regulated with hypomethylation, and 11 genes down-regulated and hypomethylated.

**Table 4:** List of genes that exhibit changes in both m^6^A methylation and mRNA level in the older group compared to younger group

Gene	pattern	chromosome	M^6^A methylation			mRNA level		
			Peak region	Peak start	Peak end	strand	Log2FC	*p*-value
FBXO16	Hyper-up	8	3’UTR	28348525	28348824	-	7.88	0.00
BUB1B	Hyper-up	15	CDS	40196615	40199635	+	2.26	0.01
GCOM1	Hyper-up	15	CDS	57708755	57709055	+	2.19	0.05
ALG11	Hyper-up	13	exon	52024458	52024758	+	1.62	0.02
AC118553.2	Hyper-up	1	3’UTR	100082036	100082246	+	1.57	0.05
TOP2A	Hyper-up	17	CDS	40392617	40398613	-	1.56	0.01
ENC1	Hyper-up	5	3’UTR	74629497	74629647	-	1.52	0.00
TTYH3	Hyper-up	7	3’UTR	2663994	2664114	+	1.30	0.01
VSIG4	Hyper-up	X	3’UTR	66021796	66022093	-	1.25	0.03
GBP4	Hyper-up	1	CDS	89186522	89188595	-	1.23	0.03
NAV1	Hyper-up	1	3’UTR	201820403	201820614	+	1.21	0.05
FOXO6	Hyper-down	1	exon	41361981	41362132	+	-4.16	0.00
TMEM60	Hyper-down	7	CDS	77793902	77794195	-	-4.12	0.00
CARD6	Hyper-down	5	CDS	40843357	40843538	+	-2.02	0.03
CHGB	Hyper-down	20	CDS	5923505	5923834	+	-1.37	0.00
FAHD1	Hyper-down	16	CDS	1827774	1827954	+	-1.13	0.04
GRAMD4	Hyper-down	22	exon	46677152	46677333	+	-1.07	0.05
SWT1	Hyper-down	1	CDS	185174473	185174951	+	-1.01	0.01
KIAA1143	Hyper-down	3	CDS	44753476	44761513	-	-0.96	0.02
PHC2	Hyper-down	1	CDS	33324821	33325001	-	-0.84	0.01
ERLIN2	Hyper-down	8	CDS	37751710	37754250	+	-0.82	0.03
NUDT16	Hyper-down	3	CDS	131383177	131383447	+	-0.75	0.03
DDR2	Hyper-down	1	3’UTR	162783533	162783714	+	-0.70	0.02
KLF13	Hyper-down	15	3’UTR	31373135	31373525	+	-0.66	0.02
SPG7	Hyper-down	16	3’UTR	89557527	89557738	+	-0.65	0.03
ZC3H13	Hyper-down	13	CDS	45970418	45979928	-	-0.60	0.03
PDF	Hypo-up	16	3’UTR	69328650	69329155	-	6.89	0.02
ZNF100	Hypo-up	19	exon	21727731	21727971	-	1.54	0.03
ARL13B	Hypo-up	3	3’UTR	94053277	94053487	+	1.48	0.01
PYGO2	Hypo-up	1	3’UTR	154958495	154958766	-	1.14	0.02
FCGR3A	Hypo-up	1	CDS	161542833	161544753	-	1.08	0.01
SGMS2	Hypo-up	4	CDS	107895406	107895886	+	1.05	0.02
ARL2BP	Hypo-up	16	3’UTR	57252258	57252528	+	1.03	0.03
NCK1	Hypo-up	3	CDS	136946219	136948511	+	0.96	0.04
SC5D	Hypo-up	11	CDS	121307317	121307467	+	0.95	0.01
A2M	Hypo-up	12	CDS	9112466	9113419	-	0.91	0.00
BMI1	Hypo-up	10	3’UTR	22329597	22329807	+	0.88	0.04
USP1	Hypo-up	1	CDS	62444767	62445155	+	0.86	0.03
EID3	Hypo-up	12	CDS	104304157	104304696	+	0.84	0.01
TCF4	Hypo-up	18	3’UTR	55224249	55224400	-	0.69	0.01
DYRK2	Hypo-up	12	CDS	67658128	67658728	+	0.68	0.02
KLHL15	Hypo-up	X	CDS	24006321	24006650	-	0.64	0.04
DNAJB6	Hypo-up	7	3’UTR	157416030	157416211	+	0.62	0.02
ABI2	Hypo-up	2	3’UTR	203439386	203439806	+	0.62	0.02
FAM208B	Hypo-up	10	CDS	5730657	5730808	+	0.60	0.01
TMF1	Hypo-up	3	CDS	69047933	69048203	-	0.59	0.02
APOB	Hypo-up	2	CDS	21026792	21026912	-	0.52	0.04
PTPMT1	Hypo-down	11	exon	47571495	47571824	+	-3.59	0.01
TNC	Hypo-down	9	CDS	115085879	115087046	-	-2.68	0.01
KLF12	Hypo-down	13	3’UTR	73694467	73694737	-	-1.59	0.00
RYR2	Hypo-down	1	CDS	237784213	237784394	+	-1.28	0.03
ZNF592	Hypo-down	15	CDS	84782922	84783103	+	-0.85	0.01
DHCR7	Hypo-down	11	3’UTR	71435159	71435340	-	-0.84	0.02
DNMBP	Hypo-down	10	CDS	99880023	99880203	-	-0.77	0.03
LATS2	Hypo-down	13	3’UTR	20974440	20974711	-	-0.69	0.01
HMGCS1	Hypo-down	5	CDS	43298614	43298885	-	-0.68	0.05
FOXP1	Hypo-down	3	3’UTR	70958795	70959066	-	-0.57	0.03
AMOTL1	Hypo-down	11	3’UTR	94869367	94871242	+	-0.44	0.03

**Fig. 4:**
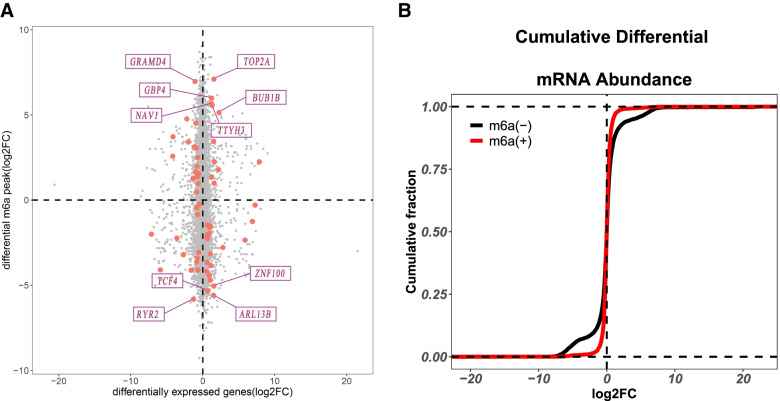
Conjoint analysis of m^6^A methylation and mRNA expression in the granulosa cells of older women. **A** Four quadrant graphs of genes with differential m^6^A methylation and differentially expressed mRNA levels. **B** Cumulative distribution curve for the gene expression changes between the two groups for m^6^A modified (red) and non-m^6^A modified (black)

The conjoint analysis of m^6^A peaks and differently expressed mRNAs were visualized, and the top 10 genes were indicated (Fig. 4A). Cumulative distribution analysis indicated that m^6^A methylated mRNAs were significantly increased in the older group compared to the younger group (Fig. 4B).

### Validation of the related mRNA

**Fig. 5:**
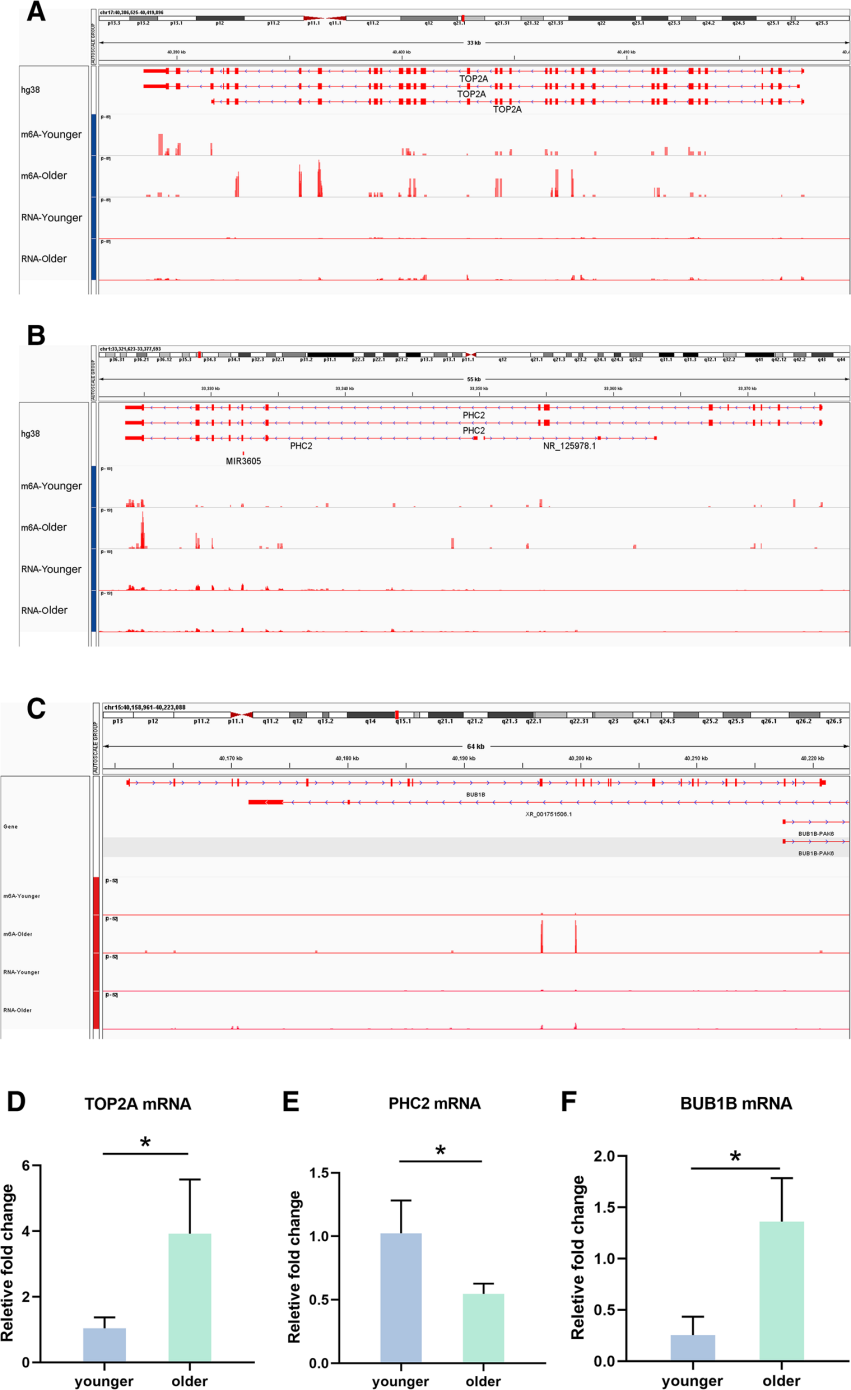
Validation of the expression of TOP2A, PHC2 and BUB1B. Integrative genomics viewer (IGV) plots of TOP2A (**A**), PHC2 (**B**), and BUB1B (**C**) m^6^A methylation abundances and expression abundances in the two groups. In the IGV plots, the peaks represent the reads signal values, and they are normalized. The maximum height of the peaks refers to read counts per million reads. For m^6^A, it refers methylation level. For RNA-seq, it refers expression level. The expression of TOP2A (**D**), PHC2 (**E**), and BUB1B (**F**) was validated in the granulosa cells using qRT-PCR

**Fig. 6:**
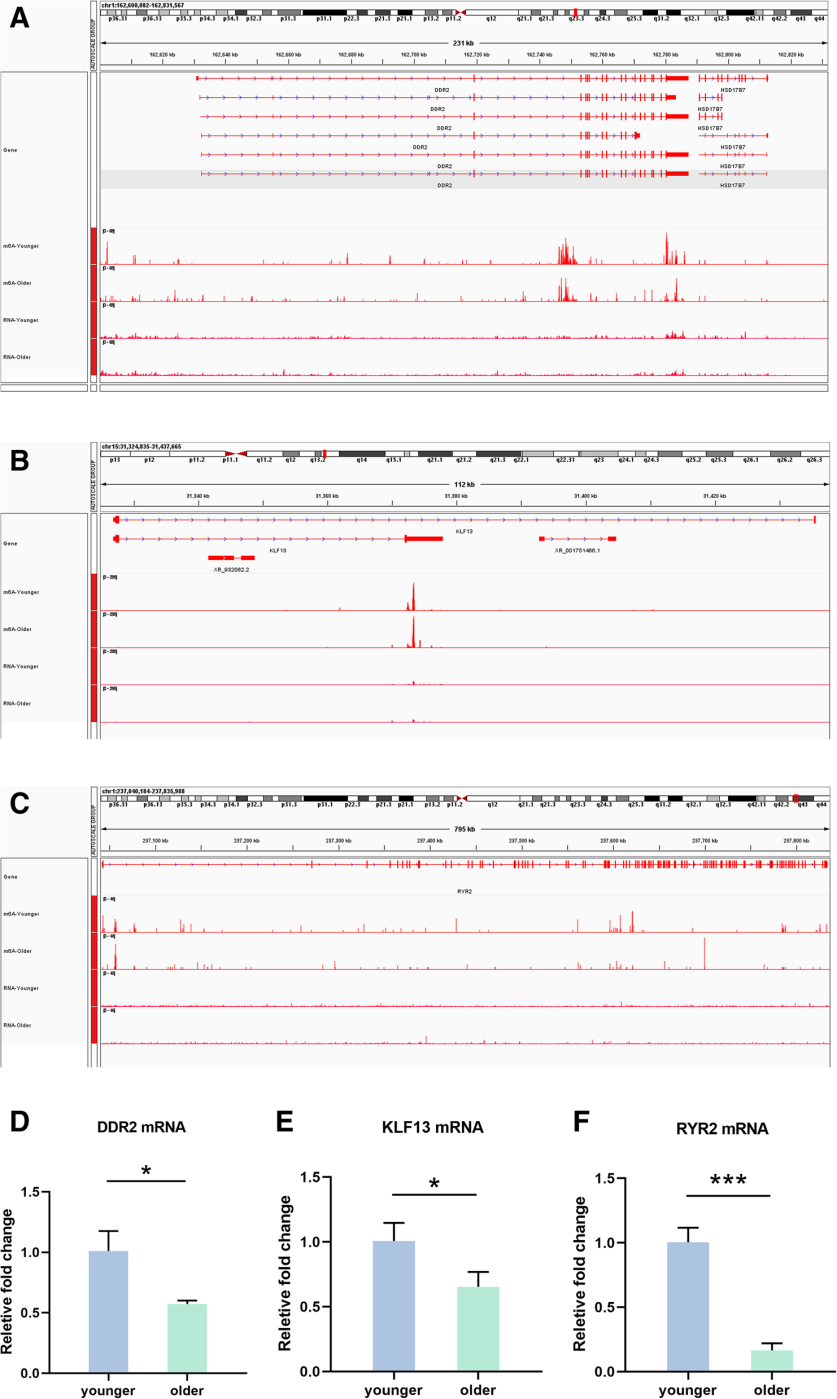
Validation of the expression of DDR2, KLF13 and RRY2. Integrative genomics viewer (IGV) plots of DDR2 (**A**), KLF13 (**B**), and RRY2 (**C**) m^6^A methylation abundances and expression abundances in the two groups. In the IGV plots, the peaks represent the reads signal values, and they are normalized. The maximum height of the peaks refers to read counts per million reads. For m^6^A, it refers methylation level. For RNA-seq, it refers expression level. The expression of DDR2 (**D**), KLF13 (**E**), and RRY2 (**F**) was validated in the granulosa cells using qRT-PCR

Among the 58 genes that had different m^6^A methylation levels and differently expressed mRNA levels, six of them (BUB1B, TOP2A, PHC2, DDR2, KLF13, and RYR2), were validated in another set of samples using qRT-PCR. Data showed that the expression of BUB1B and TOP2A were significantly up-regulated in the older group, whereas PHC2, DDR2, KLF13 and RYR2 displayed a decreased expression in this group (Figs. 5-6). The expression of these genes was consistent with the sequencing results. Integrative genomics viewer (IGV) plots of the m^6^A methylation abundances and expression abundances of these genes in the two groups were shown (Figs. 5-6).

## Discussion

M^6^A methylation is the most prevalent type of RNA methylation in eukaryotic mRNAs and plays an important role in modulating gene expression [33]. It has been well established that m^6^A methylation is essential for human fertility and is involved in the folliculogenesis and oocyte maturation [19, 34, 35]. However, the role of m^6^A methylation in ovarian aging has not been investigated. Thus, in this study, we performed MeRIP seq and RNA-Seq to characterize the transcriptome-wide m^6^A methylation in granulosa cells of older women.

RNAs can be methylated by Methyltransferase-like 3(METTL3), Methyltransferase-like 14 (METTL14) (“writers”), and demethylated by the fat mass- and obesity-associated (FTO) protein and the α-ketoglutarate-dependent dioxygenase alkB homolog 5 (ALKBH5) protein (“erasers”) [36]. There are also a bunch of “readers” of m^6^A which regulate the stability of RNA methylation, such as YTH domain proteins (YTHDF1, YTHDF2, YTHDF3, YTHDC1, and YTHDC2) [36]. Among those proteins above, many were reported to be associated with mammalian ovarian function and aging. Previous study reported that knocking down METTL3 in female oocytes inhibits its maturation and the maternal-to-zygotic transition by decreasing mRNA translation efficiency [21]. Inactivation of METTL3 in mice oocytes also leads to DNA damage accumulation in oocytes, defective follicle development, and abnormal ovulation [37]. Among readers of m^6^A methylation, studies confirmed that the protein level of FTO, but not ALKBH5, was significantly decreased in human aged ovaries and in ovaries of women diagnosed with premature ovarian insufficiency [38, 39]. Knocking down FTO, rather than ALKBH5, resulted in a decreased proliferation rate, increased apoptosis rate, and decreased cell viability in human ovarian granulosa cells [39]. YTHDC1 is required in oocyte growth and maturation and YTHDC1-deficient oocytes are blocked at the primary follicle stage [35]. YTHDC2 and YTHDF2 are also essential for the meiotic initiation and progression of female germ cells [34, 40]. In the current study, we found the number of m^6^A methylated genes was higher in the granulosa cells of older women with decreased ovarian reserve, [41]which was consistent with increased m^6^A levels that Sun et al reported [22]. Besides, our cumulative analysis also supported this point. Interestingly, the younger group displayed increased m^6^A peaks compared to the older group, indicating that granulosa cells of younger women have more m^6^A methylation sites. The overall m^6^A modification level was primarily determined by the m^6^A modified genes, as well as the level of modification of each transcript [42]. Therefore, the increased number of m^6^A peaks in the younger group does not contradict the relative lower m^6^A level in the ovaries of younger women that previous literature indicated [22]. Our study lays a foundation for further research on the expression pattern and function of epigenetic enzymes that contribute to the altered methylation in human aging ovaries.

According to our results, m^6^A methylation sites were globally enriched within the CDS and 3’UTR region in both the younger and older group. This phenomenon was in agreement with previous studies, showing CDS and 3’UTR regions were the most m^6^A modified regions in other cells and tissues [42, 43], indicating the conserveness of m^6^A methylation. A total of 3005 differently m^6^A methylated peaks were detected in the older group compared to the younger group, including 1297 hypermethylated and 1708 hypomethylated peaks. Bioinformatics analysis indicated that the most relative pathway that these dys-methylated genes enriched in was FoxO signaling pathway. Besides, several other pathways, such as adherens junction and regulation of actin cytoskeleton were also interfered with.

It has been well known that m^6^A methylation affects gene expression by regulating RNA transcription, translation, and degradation. Thus, RNA-Seq was performed to determine the transcriptome of granulosa cells from older women. Data showed that a total of 435 genes were differently expressed in the older group with 212 genes up-regulated and 223 genes down-regulated. The function and related pathways of the dysregulated genes were annotated by Go and KEGG analysis. The results indicated that mitotic nuclear division, mitotic cell cycle, and cell division were the most associated Go terms. Similarly, differently expressed genes were also enriched in the cell cycle and oocyte meiosis pathway. Previous studies demonstrated that granulosa cells in the aged antral follicles exhibited a decreased quantity, lower proliferation rate, and shorter telomere length [44, 45]. Besides, granulosa cells were proved to accelerate the aging process of oocytes [8, 10]. Combined with our results, it is legitimate to assume that the differently expressed genes might interfere with the cell cycle and contribute to the decreasing viability of granulosa cells of older women.

Conjoint analysis was performed to investigate differently expressed genes that regulated by m^6^A methylation. Data showed that a bunch of genes displayed different m^6^A methylation levels and differently expressed mRNA levels (Table 4). Most of these hyper- or hypo- methylated and differently expressed genes were reported to have a role in aging of human and other mammalian species, such as NAV1, FOXO6, CARD6, FAHD1, TCF4, DNMBP, and HMGCS 1 [46–52]. Some of the genes were confirmed to be enrolled in the process of ovarian tumorgenesis, such as FBXO16, TTYH3, VSIG4, ZC3H13, NCK1, BMI1, USP1, DYRK2, DNAJB6, TNC, KLF12, LATS2, and FOXP1 [53–65]. A small group of the genes were reported to be associated with physiological ovarian function including oocyte meiosis, oocyte maturation, ovarian insufficiency, ovarian sterol uptake and so on [66–70]. We picked up six of the genes associated with ovarian function and validated the mRNA expression by qPCR (BUB1B, TOP2A, PHC2, DDR2, KLF13, and RYR2).

Here, we found BUB1B, TOP2A, PHC2, DDR2 and KLF13 were hypermethylated and differently expressed in the granulosa cells of older women. BUB1B encoding BubR1 is functional in the spindle check during mitosis. Touati et al. reported a strong reduction of BubR1 in the ovaries of menopausal women and aged oocytes, hypothesizing that the gradual decline of BubR1 led to age-related aneuploidization [71]. Interestingly, our results indicated that BUB1B was up-regulated and hypermethylated in the granulosa cells of older women. We suspected that m^6^A methylation might be responsible for this phenomenon. It is well established that m^6^A modification is involved in almost all stages of RNA life cycle, including transcription, pre-mRNA splicing, mRNA export, mRNA stability, and translation [72]. Except for promoting mRNA degradation, m^6^A presence may block its accessibility to RNase, thereby enhancing the stability of mRNA [72, 73]. Meanwhile, m^6^A modification may also inhibit the combination of ribosomes and interfere with mRNA translation. Therefore, granulosa cells of older women presented with a hypermethylated and up-regulated expression of BUB1B without increasing Bub1R were reasonable (Table 5).

**Table 5:** Primer sequences used for gene expression analysis

Gene name	symbol	Forward primer	Reverse primer
DNA Topoisomerase II Alpha	TOP2A	GATGACAACCAGCGTGTTGAG	CCACCCAGTACCGATTCCTT
Polyhomeotic Homolog 2	PHC2	CAGAACTTGACCCTCCGAACA	GGGGAAGCCTGAGCAGTATT
BUB1 mitotic checkpoint serine/threonine kinase B	BUB1B	ACTGATAGCTGTACCCGCTGTG	GGCTTTCTGGTGCTTAGGATG
discoidin domain receptor 2	DDR2	GATGACAGCAACACTCGG	AATCACTTGGCAGGGAAA
Kruppel-like factor 13	KLF13	GTTTACGGGAAATCTTCGCA	GCGAACTTCTTGTTGCAGTC
ryanodine receptor-2	RYR2	ATAGACGGCACCATAGACA	ATGCTCAGGCGATAAAAC
Glyceraldehyde-3-phosphate dehydrogenase	GAPDH	AGAAGGCTGGGGCTCATTTG	AGGGGCCATCCACAGTCTTC

Similarly, TOP2A, which encodes a unit of topoisomerase II, was up-regulated and hypomethylated in the older group. Topoisomerase II that controls and alters the topologic states of DNA during DNA replication, transcription, and repair [74]. It has been demonstrated that genotoxic treatment increased the DNA damage accumulation in the topoisomerase II-deficient ovarian granulosa cells, suggesting a critical role of topoisomerase II in the ovary [75]. PHC2 encodes a component of polycomb group (PcG) multiprotein PRC-1like complex, maintaining the transcriptionally repressive state of numerous genes and regulating cell renewal [76]. A previous study reported that PRC1 deficiency contributes to the dysregulated expression of cytoskeletal and adherens junction proteins in the ovarian follicles [77]. Considering the functions of the altered mRNA in the older group, we suspect that m^6^A methylation reduces the expression of PHC2 expression, relating to the dysregulated adherens junctions in the granulosa cells of older women. Moreover, the expression of TOP2A and PHC2 validated by qRT-PCR also supported the sequencing data.

DDR2, discoidin domain receptor 2, is a collagen tyrosine kinase receptor gene and was down-regulated and hyper-methylated in the older group. It was reported as an important regulator of ovarian function [68]. Silencing the DDR2 resulted in decreased chromatin maintenance, disturbed hormonal signaling pathways in ovarian granulosa cells, blocked ovulation, and infertility [68]. This is in accordance with the worse fertility outcomes of aged women of both natural conception and *in-vitro* fertilization [78, 79]. KLF13 encode the Kruppel-like factors which are important Sp1-like eukaryotic transcriptional proteins [80]. It is reported to take part in the sterol uptake and steroid biosynthesis in ovarian cells [69]. Similar to DDR2, KLF13 was down-regulated and hyper-methylated in the older group according to our sequencing data. This may have a relationship with the disturbed profile of steroid hormone levels in aged women [81].

Among the six genes RYR2 (ryanodine receptor-2) was hypo-methylated in the older group. It encodes the ryanodine receptor which is one of the components of the intracellular calcium channel. This gene is selectively expressed in ovarian cumulus cells right before ovulation and plays an important role in oocyte maturation and activation [82, 83]. In our conjoint study, we found RYR2 was down-regulated and hypo-methylated in older group. This is in accordance with the decreased oocyte quality in the aged women [84].

Taken together, our study revealed the transcriptome-wide m^6^A methylation in the aged granulosa cells, and suggested this modification might be associated with the altered mRNA expression and decreased ovarian functions during aging. Therefore, our study may provide novel insights to understand the mechanisms of ovarian aging and provide a candidate reservoir to promote fertility preservation for the aging women. However, certain limitations in the study cannot be ignored. The major one is our sample size. Given the small sizes of the study groups (three samples per group), these findings should be interpreted with great caution and confirmatory studies with larger sample size are required to validate the global m^6^A methylations. Further research with a larger number of samples is required to validate the global m^6^A methylations. Besides, clarifying the role of epigenetic enzymes that contribute to the altered methylation in the aging ovaries, should be explored in subsequent studies.

## Supplementary Information

**Additional file 1.**

**Additional file 2.**

## Data Availability

The datasets generated for this study could be found in the online repositories. The names of the repositories and accession number could be found below: https://www.ncbi.nlm.nih.gov/geo/query/acc.cgi?acc=GSE181106.

## Abbreviations

m^6^A: N6-methyladenine
MeRIP-Seq: m^6^A-targeted antibody coupled with high-throughput sequencing
RNASeq: RNA sequencing
qRT-PCR: quantitative real-time polymerase chain reaction
AFC: antral follicle count
AMH: Anti-Müllerian hormone
IVF: *in vitro* fertilization
BMI: body mass index
GO: Gene ontology
KEGG: Kyoto Encyclopedia of Genes and Genomes
SD: standard deviation
