# Supplementary material for: Transcriptome-wide N6-methyladenine methylation in granulosa cells of women with decreased ovarian reserve
Source: BMC Genomics. 2022 Mar 28;23:240. doi: 10.1186/s12864-022-08462-3 (PMC8961905; doi:10.1186/s12864-022-08462-3)
Supplement: Supplementary file 1 — Additional file 1. [file 12864_2022_8462_MOESM1_ESM.docx]

Supplemental Table 1. The scheme and dose of drugs used for ovarian stimulation

|  | Yonger group | | | Older group | | |
| --- | --- | --- | --- | --- | --- | --- |
|  | YWJL^1^ | YWYQ | YQTT | OZH | OZJM | OYDX |

| Age | 27 | 25 | 27 | 45 | 43 | 45 |
| --- | --- | --- | --- | --- | --- | --- |
| Stimulation scheme | Gonadotropin  Antagonist | Gonadotropin  Antagonist | Gonadotropin  Antagonist | Gonadotropin  Antagonist | Gonadotropin  Antagonist | Gonadotropin  Antagonist |
| Drugs used for Down-  regulation | Cetrotide | Cetrotide | Cetrotide | Cetrotide | Cetrotide | Cetrotide |
| Drugs used for ovarian stimulation | Recombinant FSH | Recombinant FSH | Recombinant FSH | Recombinant FSH | Recombinant FSH | Recombinant FSH |
| Starting dose of Gn (IU) ^2^ | 300 | 200 | 225 | 375 | 300 | 300 |
| Starting day of ovarian stimulation^3^ | 3 | 3 | 2 | 2 | 2 | 2 |
| Total dose of Gn (IU) | 2175 | 1500 | 2100 | 1950 | 3075 | 2925 |
| Dose of Gn per day (IU) | 242 | 214 | 233 | 325 | 308 | 366 |
| Oral contraceptives | none | none | none | none | none | none |
| antioxidants | none | none | none | none | none | none |

1. YWJL, YWYQ, YQTT, OZH, OZJM, OYDX are labels of the six participants in the both groups. Y stands for the younger group and O stands for the older group. The rest letters are initials of each participant.
2. FSH= follicular stimulating hormone; Gn= gonadotropin; IU= international unit
3. Starting day of ovarian stimulation refers to Day N of the menstrual cycle.
