# Supplementary material for: Transcriptome-wide N6-methyladenine methylation in granulosa cells of women with decreased ovarian reserve
Source: BMC Genomics. 2022 Mar 28;23:240. doi: 10.1186/s12864-022-08462-3 (PMC8961905; doi:10.1186/s12864-022-08462-3)
Supplement: Supplementary file 2 — Additional file 2. [file 12864_2022_8462_MOESM2_ESM.docx]

**Supplemental Figures**

**Supplemental Figure 1** **The motif of m^6^A methylation in the granulosa cells.**


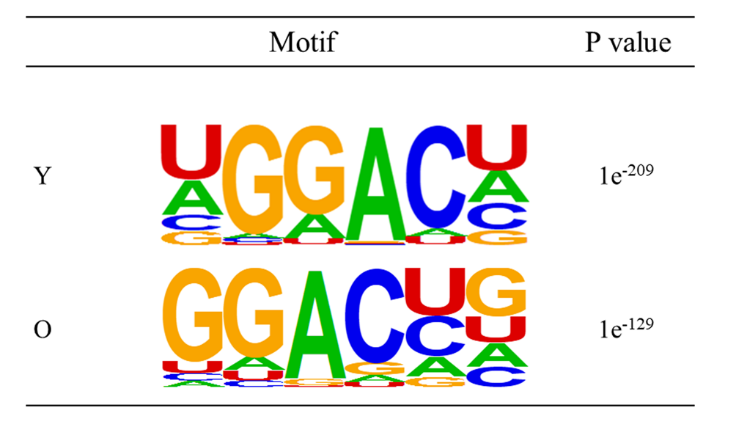


The m6A methylation motif of granulosa cells was “GGAC”.

**Supplemental Figure 2 The top 20 enriched GO terms of differentially methylated genes in the older group.**


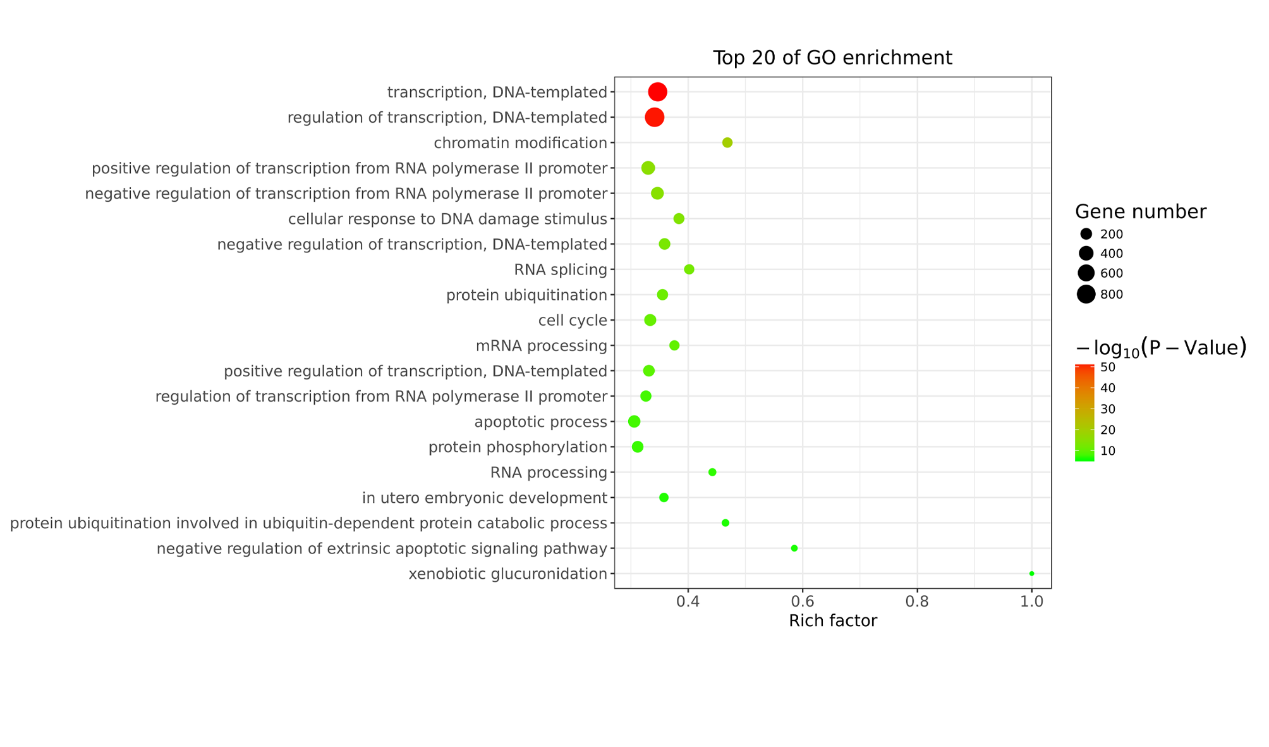


Go enrichment analysis indicated the functions of differently methylated genes of granulosa cells in the older group.

**Supplemental Figure 3** **MA-plot of gene expression changes in the granulosa cells of older women versus granulosa cells of younger women**


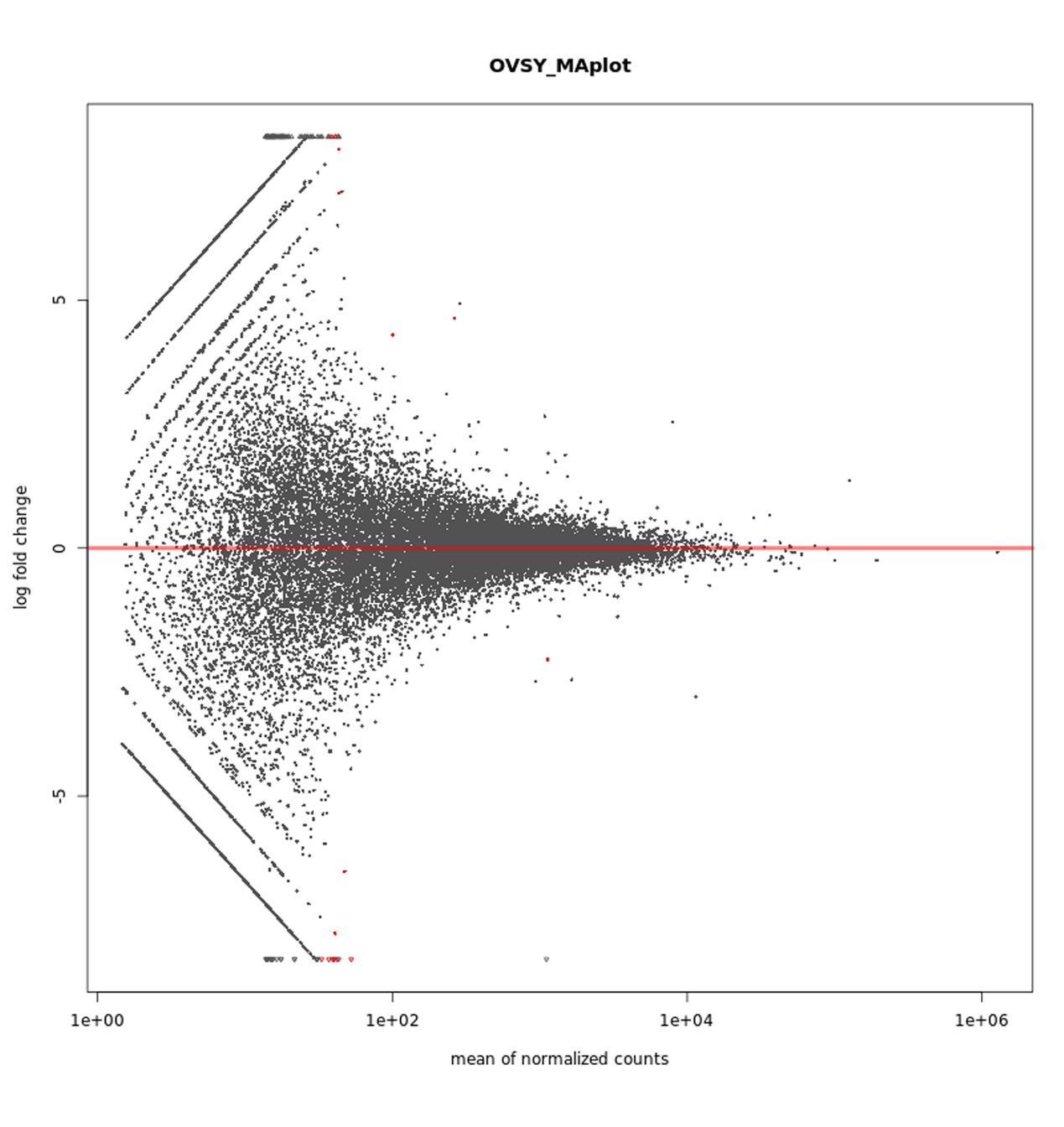


The MA plot represents the estimated log2 fold changes as a function of the mean of normalized counts.
